# Oral aripiprazole in the treatment of tic disorders in China: a cost-effectiveness analysis based on a mapping algorithm derived from a Chinese children and adolescents population

**DOI:** 10.1186/s13034-024-00786-0

**Published:** 2024-08-07

**Authors:** Chaoxin Chen, Tingting Chen, Zhongling Ke, Yi Wu, Maobai Liu, Yanhui Chen, Bin Zheng

**Affiliations:** 1 Department of Pharmacy, Fujian Medical University Union Hospital, Fuzhou, 350001 China; 2 Department of Pharmacy, Fujian Obstetrics and Gynecology Hospital, Fuzhou, 350001 China; 3 Department of Pediatric, Fujian Medical University Union Hospital, Fuzhou, 350001 China; 4 School of Pharmacy, Fujian Medical University, Fuzhou, 350004 China

**Keywords:** Tic disorders, Cost-effectiveness, Mapping, Aripiprazole, Children

## Abstract

**Background:**

Oral aripiprazole exhibits favorable clinical efficacy and safety in the suppression of tics in children and adolescents with tic disorders. This study aims to evaluate and compare the cost-effectiveness of high-dose and low-dose aripiprazole in children and adolescents with tic disorders from the perspective of the Chinese healthcare system.

**Methods:**

A questionnaire survey was conducted on 146 patients with tic disorders, of whom 144 completed EQ-5D-Y and YGTSS. Four models were built to convert YGTSS onto EQ-5D-Y utility using two mapping algorithms. We constructed a decision tree model containing efficacy and safety to compare the cost-effectiveness of high-dose and low-dose aripiprazole based on our mapping function.

**Results:**

The GLM with model 1 (YGTSS total tic scores) was selected as the preferred function in our decision tree model. The base case cost-effectiveness analysis showed that compared to low-dose aripiprazole, high-dose aripiprazole improves effectiveness by 0.001QALYs and increases the overall cost by $197.99, resulting in an ICER of $174339.22 per QALY, which exceeds three times the gross domestic product per capita. Hence, high-dose aripiprazole is not likely to be a cost-effective option for child patients with tic disorders. One-way sensitivity analysis and probabilistic sensitivity analysis showed that these results is robust.

**Conclusion:**

On the basis of currently available data, low-dose aripiprazole may be a safe, effective, and economical dosage for children and adolescents with tic disorders.

**Limitations:**

The main limitation of our study is the lack of utility directly used for cost-effectiveness analysis. We obtained the utility of patients with tic disorders indirectly by the mapping function. This may introduce some bias and uncertainty. And it is a limitation to use the direct medical costs of Germany in our model. Although we converted it to the equivalent value of China using purchasing power parities, caution should be exercised when interpreting the results of this study.

## Introduction

Tics are defined as sudden, rapid, recurrent and non-rhythmic motor movements or vocalizations. Tic disorders including Tourette Syndrome (TS), Chronic motor or vocal Tic Disorder (CTD), Transient Tic Disorder (TTD) and tic disorder not otherwise specified  [1] . Tic disorders is a neuropsychiatric spectrum disorder usually onset in childhood at the age of 5 to 6 years, peaking at the age of 10 to 12 years, and most individuals experience markedly reduced numbers of tics or are free of tic in early adulthood [2–4]. The prevalence of tic disorders in childhood and adolescents is 1.15% [5]. Tics have a negative impact on the quality of life (QoL) of children and adolescents. Some studies [6–8] denoted that the health-related quality of life (HR-QoL) of tic patients is lower than health children, especially in the domain of social, academic, family and psychological [9–11]. Furthermore, Jalenques et al. found that the parents of children with TS were more susceptible to mental disorder [12]. The European Society for the Study of Tourette syndrome recommended that drugs treatment should be considered in the following condition, especially when persisting for some days: Tics cause subjective discomfort, sustained social problems, social and emotional problems or functional interference [13]. Therefore, it is essential to carry out drug or non-drug intervention for patients with TS to reduce the economic and caregiver burden. A burden of disease research in Germany showed that the annual TS-specific costs totalled 3404€ (costs were in year 2006–2007 values) [14]. The cost-effectiveness of non-drug intervention for the treatment of TS has been evaluated in many studies [15–17]. For instance, Guliani et al. [15] compared the cost-effectiveness of Internet-delivered Cognitive Behaviour Therapy support once-weekly (1WS), the results showed that 1WS could be an economically attractive Cognitive Behaviour Therapy for TS. But there was no research to estimate the economic of drugs for treating TS. Herein, we performed a cost-effectiveness analysis to estimate the economic of two different doses of oral aripiprazole for treating tic disorders based on a mapping algorithm derived from a Chinese children and adolescents population.

## Methods

### Derivation of the mapping functions

#### Population

Children and adolescents with tic disorders who visited in Fujian Medical University Union Hospital from 2018 to 2021 were invited to participate in our survey. Participants could be diagnosed as tic disorders. The study was approved by the institutional review boards.

Inclusion criteria: (1) Male or female child or adolescent, ≤ 17 years of age at the time of signing the informed consent/assent. (2) Diagnosis of TS that met the Chinese Classification of Mental Disorders-third edition (CCMD-3). (3) Patient and designated guardian(s) able to comprehend and comply with protocol requirements. (4) Patient and designated guardian(s) volunteered to participate in this study and written informed consent was obtained.

#### Measurements

All patients received an informed consent informing them about the study and asking for their voluntary participation. Those who agreed to participate were provided with the Yale Global Tic Severity Scale (YGTSS) [18, 19] and the EuroQol five-dimension questionnaire youth version (EQ-5D-Y) [20], along with some questions regarding sociodemographic characteristics. The questionnaires were evaluated by the caregiver. In addition, the sociodemographic characteristics data and questionnaires was distributed and collected by trained personnel.

The YGTSS is widely used to assess the severity of tic disorders, and its internal consistency and test-retest raliability have been verified [18]. The YGTSS includes three subscales: motor tics, vocal tics and a separate impairment scale. Motor and vocal were included in our survey. The motor and vocal tics are rated separately on a 0–5 scale across five dimensions: number, frequency, intensity, complexity and interference [18]. The scores can be summed to produce the Total Motor Tic score (range 0–25) and the Total Vocal Tic score (range 0–25), and the combined Total Tic score (range 0–50) [18].

The EQ-5D is the most widely used preference-based scale for calculating health-related quality of life [21, 22]. EQ-5D-Y is a version specifically for children and adolescents. The EQ-5D-Y measures the health state (such as 11,111, 21,231 etc.) of patients from five dimensions including mobility, self-care, performance of usual activities, pain or discomfort, and anxiety or depression. Then, the utility index can be calculated by the value set and we adopted the EQ-5D-Y-3 L Value Set for Chinese population [23].

#### Statistical analysis

To describe the sample, we used frequencies and percentages for categorical variables and means and standard deviations (SDs) for quantitative variables. Unless critical information was missing, all data was used for developing the mapping functions and validating these functions.

##### Mapping algorithms

We used two different utility mapping algorithms to convert YGTSS onto the EQ-5D-Y-3 L.

1. General linear models (GLMs). A GLM requires the dependent variable is continuous and the residuals must be normally distributed. However, the EQ-5D-Y utility are defined in an interval, so GLM is not always appropriate [24].
2. Beta regression models. To avoid above issue, we built beta regression models, which use the logit function as a link and allow modeling outcomes with skewed distributions [25]. The EQ-5D-Y-3 L is a three-level scale, which is prone to complete health states (11,111), resulting in a utility value of 1, which is called the “ceiling effect”. The “ceiling effect” makes the data tend to be skewed and with truncated tails (censored data). The beta regression models require the response variable has to be restricted to the open interval (0, 1); therefore, we transformed the boundary points of the EQ-5D-Y utility to slightly lower or higher values by applying the formula *[Y(N-1) + 0.5]/N*, where *Y* is the observed EQ-5D-Y utility, *N* is the number of participant in our survey.

Four models strategy were developed used in the 2 statistical approaches. The following predictor variables were considered: Model 1: the YGTSS Total tic scores; Model 2: the YGTSS subscales; Model 3: the YGTSS Total tic scores plus covariates; Model 4: the YGTSS subscales plus covariates. Sex, age, height and weight were included as covariates. As a dichotomous variable, sex was assumed to be 0 for females and 1 for males.

The Akaike (AIC) and the Bayesian (BIC) information criteria and adjusted R-squared for GLM and beta models were calculated to compare the goodness-of-fit. We also compare the predictive performance of the 4 models by calculated the mean absolute error (MAE) and the root mean squared error (RMSE). All statistical analyses were conducted with R softwre version 4.1.2.

### Cost-utility analysis

#### Model structure

**Fig. 1:**
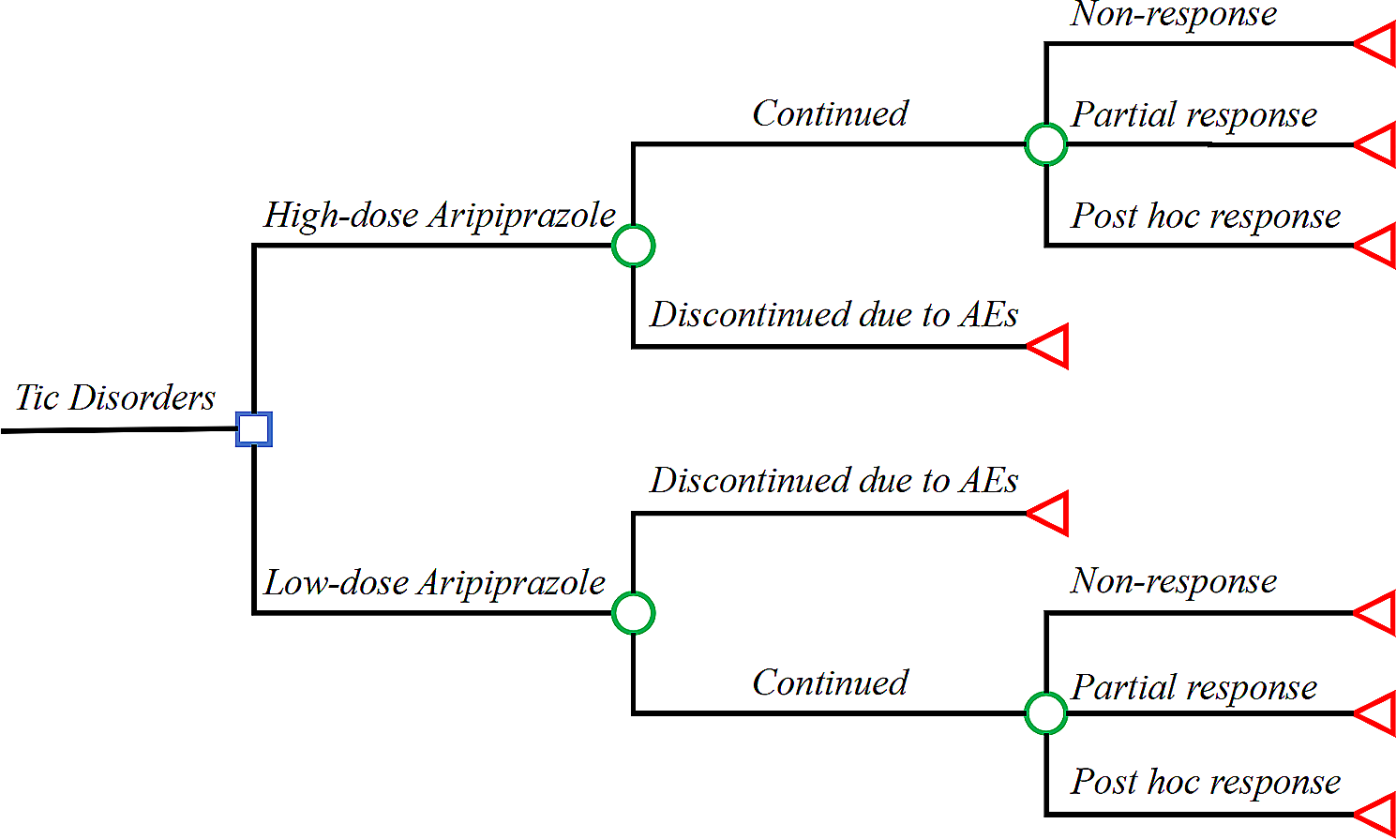
Schematic representation of model structure. A decision-tree analysis assessed aripiprazole as monotherapy management of Tourette disorders over a time horizon of 52 weeks

A decision tree model was constructed to simulate the clinical management of children and adolescents with tic disorders for 1 year (52 weeks) under two different doses of aripiprazole and placebo treatment, using TreeAge Pro Version 2022 (Fig. 1). The clinical events included in the model consist of adverse reactions leading to treatment discontinuation, as well as the efficacy of the drug after administration: post hoc response, partial response, and non-response. Post hoc response, partial response, and non-response were defined as a reduction in the YGTSS total score of > 50%, 25–50%, and < 25% from baseline, respectively [26].

#### Model input

From the perspective of China’s healthcare system, this study only examined direct medical costs, including the cost of drugs, outpatient care, inpatient stay, rehabilitation, office-based physicians, ancillary therapy and auxiliary material/sundries, as shown in Table 1. There were no more research reporting the direct medical costs from the perspective of China’s healthcare system. Consequently, all cost data origined from the research of *Dodel et al.*, and they were converted by the equivalent value of China using purchasing power parities (PPPs) [14]. Then, the costs were converted based on the average exchange rate of the US dollar in December 2022 (USD 1 = 6.98 RMB), with a discount rate of 5%. The unit price of aripiprazole in China was obtained from the *Yaoyuan network (XXX) *[27]. The treatment doses of aripiprazole were chosen based on the research by *Floyd Sallee* et al. Therefore, the doses of children and adolescents with tic disorders were 5 mg/day (Low dose) or 10 mg/day (High dose).

**Table 1:** Parameter input

Parameter	Baseline value	Distribution	Range	Source
Direct medical costs	$ 31.50	Gamma	25.20–37.80	Dodel et al. [12]
Outpatient care	$ 1.14	Gamma		
Inpatient stay	$ 15.92	Gamma		
Rehabilitation	$ 8.03	Gamma		
Office-based physicians	$ 2.17	Gamma		
Ancillary therapy	$ 4.22	Gamma		
Auxiliary material/sundries	$ 0.02	Gamma		
Cost of aripiprazole	$ 0.72/5 mg	Gamma	0.32–1.52	
Discontinuation rate due to AEs				Sallee et al. [23]
Low-dose aripiprazole	0.023	Beta	0.021–0.025	
High-dose aripiprazole	0.156	Beta	0.140–0.172	
Placebo	0.023	Beta	0.021–0.025	
Probability of post hoc response				Sallee et al. [23]
Low-dose aripiprazole	0.405	Beta	0.365–0.446	
High-dose aripiprazole	0.571	Beta	0.514–0.628	
Placebo	0.167	Beta	0.150−0.184	
Probability of partial response				Sallee et al. [23]
Low-dose aripiprazole	0.333	Beta	0.300–0.366	
High-dose aripiprazole	0.314	Beta	0.283–0.345	
Placebo	0.548	Beta	0.493–0.603	
YGTSS total tic scores	30.37	Gamma	24.30–36.44	*S*allee et al. [23]
Health utilities				
Utility of post hoc response	0.934			
Utility of partial response	0.919			
Utility of non-response	0.902			
Disutility of AEs	−0.090	Beta	−0.045 to −0.135	Assumed

The utility of children and adolescent with tic disorders before and after treatment were converted based on the research of Floyd Sallee et al*.* using our mapping model [26]. The probability of post hoc response, the probability of partial response and the discontinuation rate due to AEs were also originated from the research of Floyd Sallee et al. [26]. We assumed that the disutility of adverse reactions leading to treatment discontinuation was 10% of the initial utility.

#### Sensitive analysis

The uncertainties of key parameters were analyzed using one-way sensitivity analysis (OWSA) and probabilistic sensitivity analysis (PSA). For drug costs and direct medical costs, one-way sensitivity analysis were performed across a wide range (± 20%) to capture all possible scenarios. The range of probability was ± 10%. For all key parameters, PSA was applied to reflect the impact of their stochastic characteristics on the results. In the PSAs, we performed 1000 Monte Carlo iterations on the uncertainty of all key parameters within 95% confidence intervals. Reasonable values were used in the absence of these confidence interval values (e.g., 20%). The ranges and distributions of the parameters used in the sensitivity analyses are given in Table 1. According to the WHO’s recommendation, three times China’s per capita GDP in 2022 was used as the threshold value (36832.95 US dollars).

## Results

We included 146 patients who met the selection criteria and agreed to participate. Of these, 144 (98.6%) completed EQ-5D and YGTSS (2 incomplete, 1 for EQ-5D and 1 for YGTSS), but there were some questionnaires missing the data of height (*n* = 3) and weight (*n* = 4). All of 144 patients was included for developing the mapping functions and validating these functions. The sociodemographic and clinical data were shown in Table 2.

**Table 2:** The characteristics of the patients

Parameter	Number	Ratio, %	Mean (SD)
Sex			
Men	127	86.99	
Women	19	13.01	
Age (years)	146		9.01 (2.47)
Height (cm)	143		139.05 (14.50)
Weight (kg)	142		34.49 (11.92)
Comorbidities			
Neuropsychiatric disorders	8	6.16	
Other co morbidities	12	8.22	
YGTSS			
YT	145		16.24 (8.73)
YM	145		11.28 (5.07)
YV	145		4.95 (6.36)
EQ−5D-Y utility	145		0.9312 (0.0832)

Neuropsychiatric disorders, included two of epilepsy, two of attention-deficit/hyperactivity disorder and four of other neuropsychiatric disorders (unknown); Other co morbidities, inclued eight of rhinitis, one of enuresis, three of others (unknown); YT, YGTSS Total scores; YM, YGTSS Motor scales; YV, YGTSS Vocal scales

### Derivation of the mapping functions

Table 3 reported the relationship between the EQ-5D-Y utilities and YGTSS scores. We first used the GLM and Beta regression to convert YGTSS into the EQ-5D-Y, the results shown that it has statistically significant. When including sex, age, height and weight in the predictive models, only sex has statistically significant (*P* < 0.05). In the GLM regression and beta regression, the best goodness of fit and the best predictive accuracy was found in model 3, yielded lower AIC and BIC, higher adjusted R^2^. And the predicted values of model 3 was closer to the observed values.

**Table 3:** Fit measures for the different models used to predict the EQ-5D-Y utilities based on YGTSS scores. (*n* = 144)

	Models without demographics		Models with demographics	
	Model 1 Scale total scores	Model 2 Subscale scores	Model 3 Model 1 plus demographics with *P* < 0.05	Model 4 Model 2 plus demographics with *P* < 0.05
**GLM**				
Variables	YT	YM + YV	YT + Sex	YM + YV + Sex
Parameters, β (SE)				
Intercept	0.9881 (0.0139)	0.9856 (0.0164)	1.0201 (0.0220)	1.0179 (0.0238)
YT	−0.0035 (0.0008)		−0.0036 (0.0008)	
YM		−0.0032 (0.0013)		−0.0033 (0.0013)
YV		−0.0037 (0.0010)		−0.0038 (0.0010)
Sex			−0.0357 (0.0191)	−0.0356 (0.0191)
Predicted index				
Mean (SD)	0.9313 (0.0302)	0.9313 (0.0303)	0.9313 (0.0325)	0.9313 (0.0327)
Range	0.8410 to 0.9881	0.8404 to 0.9856	0.8346 to 0.9988	0.8341 to 0.9982
Fit measures				
AIC	−321.80	−320.04	−323.34	−321.56
BIC	−312.89	−308.16	−311.46	−306.71
Adjusted R^2^	0.125	0.120	0.140	0.135
Predictive accuracy				
MAE	0.0599	0.0601	0.0583	0.0585
RMSE	0.0775	0.0775	0.0766	0.0765
**Beta**				
Variables	YT	YM + YV	YT + Sex	YM + YV + Sex
Parameters, β (SE)				
Intercept	3.0751 (0.1661)	3.1013 (0.1963)	3.4553 (0.2696)	3.5025 (0.2913)
YT	−0.0281 (0.0088)		−0.0290 (0.0087)	
YM		−0.0314 (0.0156)		−0.0342 (0.0156)
YV		−0.0259 (0.0123)		−0.0256 (0.0122)
Sex			−0.4149 (0.2357)	−0.4206 (0.2358)
Predicted index				
Mean (SD)	0.9304 (0.0175)	0.9304 (0.0176)	0.9303 (0.0193)	0.9302 (0.0194)
Range	0.8694 to 0.9559	0.8697 to 0.9569	0.8610 to 0.9638	0.8613 to 0.9643
Fit measures				
AIC	−533.58	−531.70	−535.02	−533.22
BIC	−524.67	−519.82	−523.14	−518.37
Adjusted R^2^	0.101	0.094	0.112	0.105
Predictive accuracy				
MAE	0.0596	0.0595	0.0586	0.0585
RMSE	0.0775	0.0775	0.0768	0.0768

AIC, Akaike information criterion; BIC, Bayesian information criterion; GLM, general linear model; YT, YGTSS Total scores; YM, YGTSS Motor scales; YV, YGTSS Vocal scales; SE, standard error; SD, standard deviation; MAE, mean absolute error; RMSE, root mean square error

Adding sex to Model 1 in GLM, the* P* value for sex was* P *= 0.0630

Adding sex to Model 2 in GLM, the *P* value for sex was* P* = 0.0649

Adding sex to Model 1 in beta model, the *P* value for sex was *P* = 0.0784

Adding sex to Model 2 in beta model, the P value for sex was *P* = 0.0745

### Cost-utility analysis

#### Conversion of utility

We didn’t include sex in our decision tree model, therefore, a GLM with model 1 was considered the preferred model to convert YGTSS total scores to utilities (Eq. 1).

1 $$\text{Predicted EQ-5D-Y utility index} = 0.9881-0.0035 \times YT$$

#### Base case analysis

The decision tree model was used to predict the costs and health outcomes of high-dose and low-dose aripiprazole (Table 4). The benefit of children and adolescents with tic disorders receiving high-dose aripiprazole is 0.910QALYs, which is 0.001QALYs more than those receiving low-dose aripiprazole. Compared to children and adolescents receiving low-dose aripiprazole, the incremental cost of receiving high-dose aripiprazole is $197.99, resulting in an ICER of $174339.22 per QALY, which exceeds the acceptable threshold (36832.95 US dollars, three times China’s per capita GDP in 2022). In the patients receiving high-dose aripiprazole, there are 747 response per 1000 patients, which is 26 patients more than those receiving low-dose aripiprazole.

**Table 4:** Costs and health outcomes over 52 weeks in China

	Costs in USD (per patient)	Incremental cost (per patient)	QALYs (per patient)	Incremental QALYs (per patient)	ICERs	Partial response cases (per 1000 patients)	Post hoc response cases (per 1000 patients)
Low-dose aripiprozole	1894.98	–	0.909	–	–	325	396
High-dose aripiprozole	2092.97	197.99	0.910	0.001	174339.22	265	482

#### Sensitive analysis

**Fig. 2:**
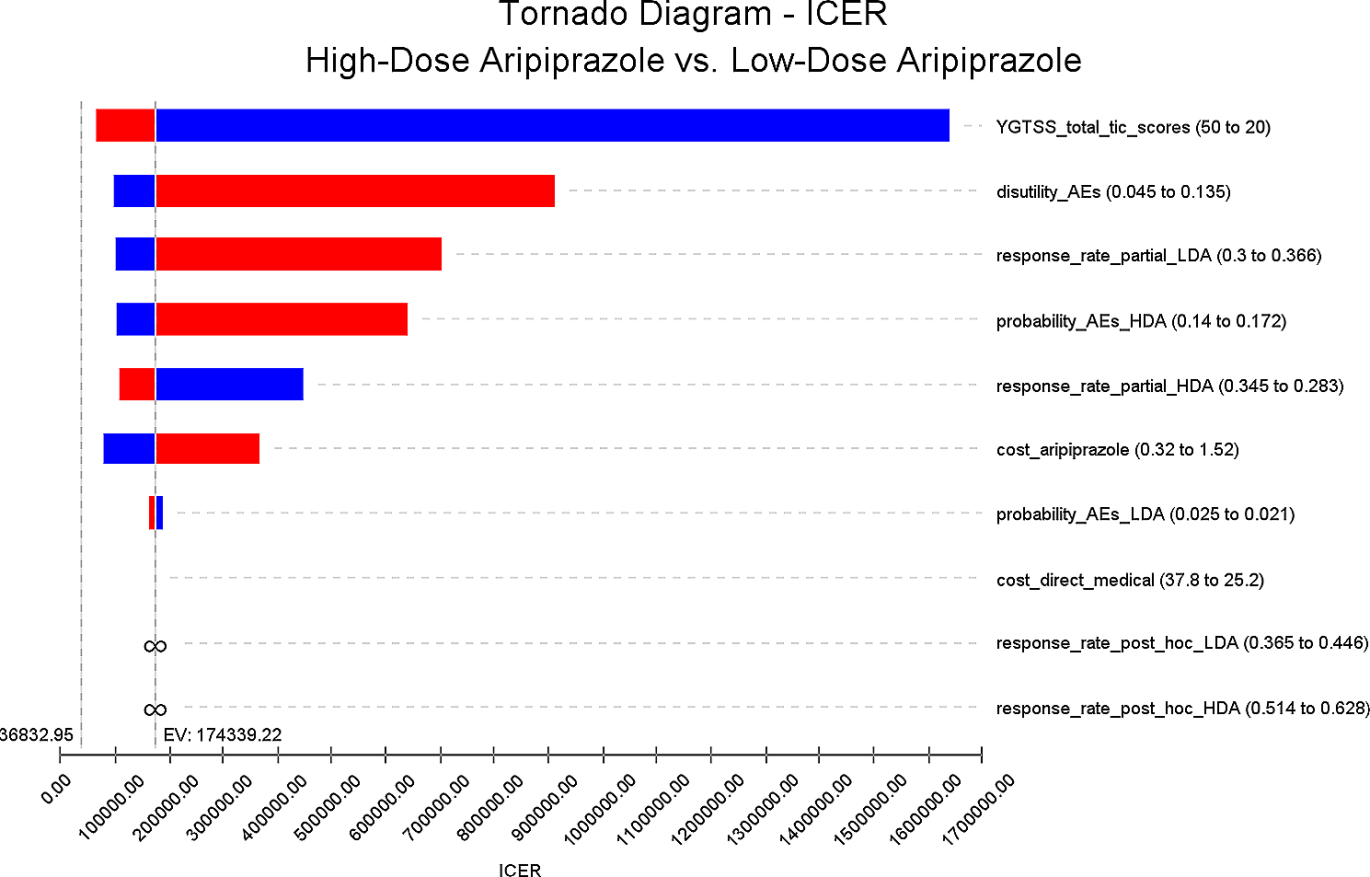
The Tornado Charts (ICER) of high-dose aripiprazole vs. low-dose aripiprazole

**Fig. 3:**
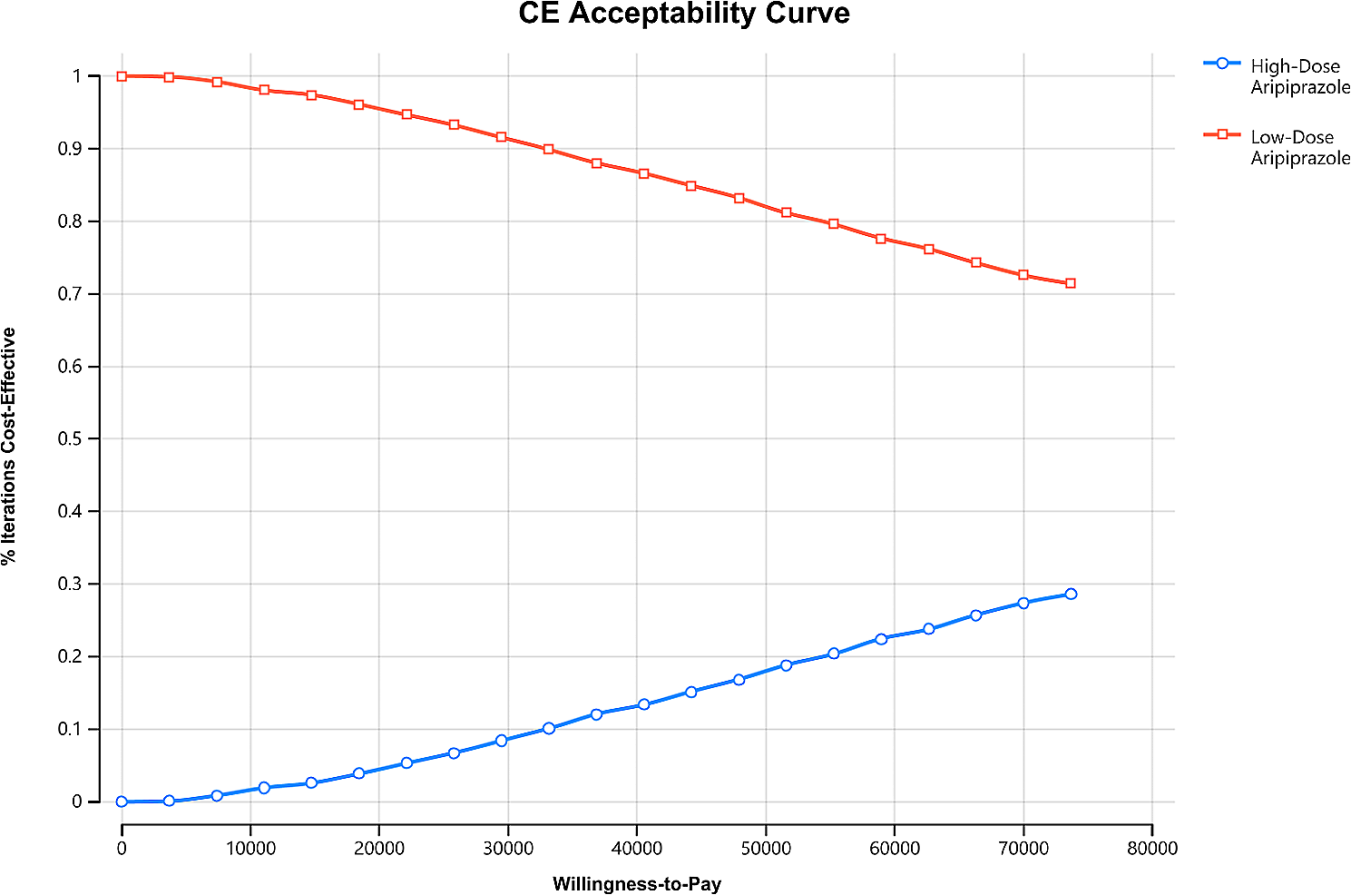
The acceptability curves of high-dose aripiprazole vs. low-dose aripiprazole

**Fig. 4:**
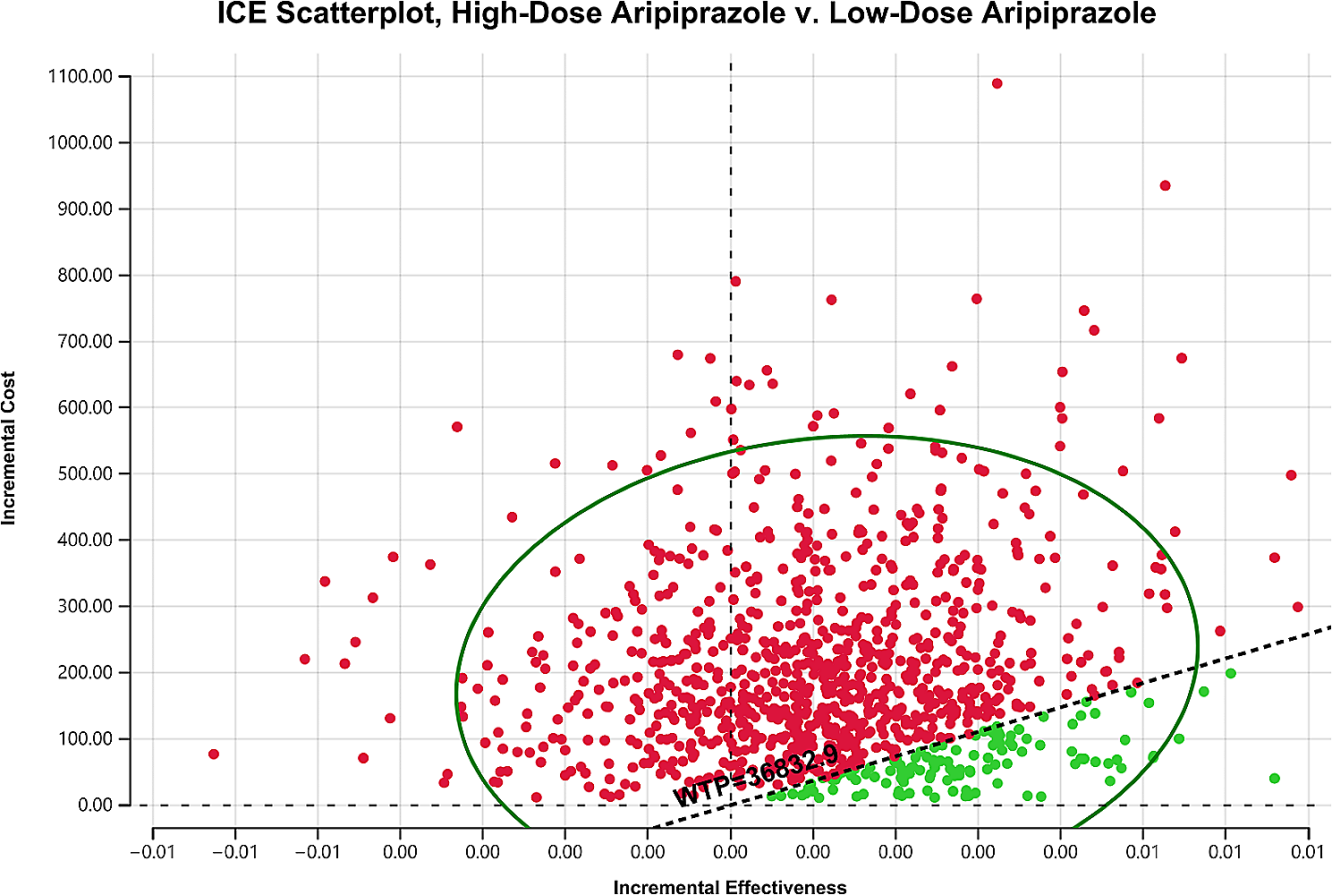
The scatter plots of high-dose aripiprazole vs. low-dose aripiprazole

One-way sensitivity analysis was used to verify the stability of the results (Fig. 2). Some parameters cause fluctuations in ICER, the most influential parameters are YGTSS total tic scores, the disutility of adverse reactions leading to treatment discontinuation and the partial response rate of low-dose aripiprazole. However, the variation of ICER are all above the WTP. In other words, the results are robust. The acceptability curve revealed that compared to low-dose aripiprazole, as WTP threshold increases, the probability of the high-dose aripiprazole strategy being cost-effective gradually increases. At a WTP threshold of $36832.95 per QALY, the probability of the low-dose aripiprazole strategy being cost-effective is 87.3% (Fig. 3). Incremental cost-effectiveness scatterplot showed that most of the scatter points were above the threshold line (Fig. 4).

## Discussion

The atypical antipsychotic aripiprazole, a dopamine D2- and serotonin 5-hydroxytryptamine (5-HT)1 A receptor partial agonist and 5-HT2A receptor antagonist, has been approved by the FDA for the treatment of tic disorders. As is well-known, rational administration of drug encompasses aspects of safety, efficacy and economy. Buts current researches on aripiprazole in tic disorders focus more on safety and efficacy. For instance, the efficacy and safety of two different doses of aripiprazole for the treatment of tic disorders in children and adolescents was demonstrated in a randomized, double-blind, placebo-controlled trial [26]. Compared with low-dose aripiprazole, high-dose aripiprazole is more effective, but cause more serious adverse reactions, potentially leading to more expensive medical costs [26]. In a newly published systematic review and network meta-analysis [28], aripiprazole outperformed placebo and clonidine in the treatment of TS, with moderate certainty of evidence. But in terms of tolerability and acceptability, there were no relevant findings for any of the efficacious medication, with low to very low certainty of evidence. In contrast, little attention has been paid to the economy of aripiprazole for TS.

In this study, we evaluated the cost-effectiveness of high-dose and low dose aripiprazole for the treatment of children and adolescents tic disorders. Our results found that compared to low-dose aripiprazole, high-dose aripiprazole improves effectiveness by 0.001QALYs and increases the overall cost by $197.99, resulting in an ICER of $174339.22 per QALY, which exceeds the WTP threshold. One-way sensitivity analyses using ± 20% as a range boundary revealed that the main driver of ICER is the YGTSS total tic scores. In other words, the main driver of ICER is the utility of patients with TS. However, the relationship between the ICERs and thresholds remained unchanged no matter lowered or upped values of key parameters. PSA indicated that low-dose aripiprazole may be more cost effective than high-dose aripiprazole. These findings reveal that low-dose aripiprazole maintenance therapy is suitable for use in clinics when price, safety and efficacy are taken into account simultaneously.

For all we know, this is the first report to build decision tree model to estimate the economic of drugs for treating tic disorders based on a mapping algorithm derived from a Chinese children and adolescents population. Our model not only considers the efficacy of the drug, but also the adverse reactions, making our model more in line with the clinical process. In our decision tree model, we calculated the health utilities of patients with TS. A strength of our research was the use of multiple statistical methods which enabled us to evaluate and select the best-performing algorithm, while also considering convenience in use.

The changes of YGTSS total tic scores is widely used as an outcome in clinical trial of tic disorders. However, to our knowledge, there was no report to map the disease-specific YGTSS measure onto the generic preference-based EQ-5D-Y. Hence, our research made an attempt to map YGTSS total tic scores onto EQ-5D-Y utilities using two different algorithms. We have obtained mapping functions with an acceptable predictive performance. More importantly, it provides a method of converting the utilities for use in cost-utility studies when utilities are not available.

The population of this research are children and adolescents, therefore, we use the EQ-5D-Y to derive mapping functions. Compared with the original version of EQ-5D, it is more likely to be understood by children and adolescents. Another advantage of our study is that two different algorithms were used to convert YGTSS total tic scores and YGTSS subscale scores to EQ-5D-Y utilities. The results show that both the subscale scores and the total scores are correlated with EQ-5D utilities. Furthermore, our models consider sociodemographic variables such as age, sex, height and weight, concluding that the model improved, although only sex was significant.

This study has some limitations. The main limitation of our study is the lack of utility directly used for cost-effectiveness analysis. We used a popular method, mapping function, to indirectly obtain the health utilities before and after treatment of patients with tic disorders. This may lead to the health utilities that are slightly different from the actual situation for patients with TS, although we used two algorithms for optimization. Thus introducing some biases and uncertainty. In addition, we assumed the disutility of adverse reactions was converted based on the initial utility of tic disorders. This may introduce some bias and uncertainty. Secondly, because there are no reports to estimating an EQ-5D-Y-5 L value set for China, this study used EQ-5D-Y-3 L not the EQ-5D-Y-5 L. Compared with EQ-5D-Y-5 L, EQ-5D-Y-3 L have a more obvious ceiling effect, which may affect the predict performance of the model. Thirdly, it is a limitation to use the direct medical costs of Germany in our model. Although we converted it to the equivalent value of China using purchasing power parities (PPPs), caution should be exercised when interpreting the results of this study. Fourthly, there are several drug options that a child or adolescent with tic disorders could receive, but our research only considered aripiprazole as a comparator, additional treatment strategies were not included. We will include other treatment strategies for comparison when new high quality clinical trials are published. Fifthly, the samples we collected are too few to conduct internal validation and external validation, which is not comprehensive for evaluating the performance of the model.

## Conclusions

From the perspective of China’s healthcare system, although patients had a higher response rate to high-dose aripiprazole, high-dose aripiprazole does not appear to be a cost-effective treatment for children and adolescents with tic disorders at a WTP threshold of $36,832.95 per QALY. These findings reveal that low-dose aripiprazole is a suitable treatment options in clinics when price, safety and efficacy are taken into account simultaneously. Thus, the waste of healthcare costs and resource allocation can be reduced in China. Meanwhile, further research or clinical trials using health utilities as a outcome have to be carried out based on the limitations of the current study.

## Data Availability

The data arenot publicly available due to privacy orethical restrictions.
